# Supplementary material for: Decrease in Ambient Fine Particulate Matter during COVID-19 Crisis and Corresponding Health Benefits in Seoul, Korea
Source: Int J Environ Res Public Health. 2020 Jul 22;17(15):5279. doi: 10.3390/ijerph17155279 (PMC7432095; doi:10.3390/ijerph17155279)
Supplement: Supplementary file 1 [file ijerph-17-05279-s001.pdf]

Table S1. Concentration-response functions used in the study.

Relative risk per 10 μg/m^3 increase of $PM_{2.5}$	Relative risk	95% lower confidence interval	95% upper confidence interval
MCC study			
Non-accidental all-cause mortality	1.0068	1.0059	1.0077
Cardiovascular disease mortality	1.0055	1.0045	1.0066
Respiratory disease mortality	1.0074	1.0053	1.0095
Seoul study			
Non-accidental all-cause mortality	1.0033	1.0001	1.0066
Cardiovascular disease mortality	1.0076	1.0012	1.0141
Respiratory disease mortality	1.0177	1.0055	1.0301

Table S2. Estimated number of mortalities attributable to $PM_{2.5}$ exposure from January to April in each year from 2016 to 2020

Year	$PM_{2.5}$ (μg/m^3)	Non-accidental mortality (N)	Cardiovascular mortality (N)	Respiratory mortality (N)
MCC study				
2016	28.1	237.9 (206.7, 268.9)	43.1 (35.3, 51.6)	28.8 (20.7, 36.8)
2017	31.7	260.4 (226.3, 294.3)	49.9 (40.9, 59.7)	31.2 (22.5, 39.9)
2018	30.6	273.4 (237.7, 309.1)	50.9 (41.7, 60.9)	36.8 (26.5, 47.1)
2019	34.6	293.5 (255.1, 331.7)	54.7 (44.9, 65.5)	36.9 (26.5, 47.1)
2020	25.6	211.4 (183.7, 239.0)	39.4 (32.3, 47.2)	26.6 (19.1, 34.0)
Seoul study				
2016	28.1	116.1 (3.5, 230.9)	59.4 (9.5, 108.9)	67.6 (21.5, 112.5)
2017	31.7	127.2 (3.9, 252.8)	68.6 (11.0, 125.8)	73.3 (23.3, 121.7)
2018	30.6	133.6 (4.1, 265.5)	70.0 (11.2, 128.4)	86.4 (27.5, 143.5)
2019	34.6	143.5 (4.4, 284.9)	75.3 (12.0, 137.8)	86.3 (27.5, 143.2)
2020	25.6	103.2 (3.1, 205.2)	54.2 (8.7, 99.6)	62.5 (19.8, 104.2)

Table S3. The daily average amount of vehicles entering the Seoul Metropolitan Area highways and number of citizens who used subways of Seoul Metropolitan area from January to April each year from 2016 to 2020

	Year 2016	Year 2017	Year 2018	Year 2019	Year 2020
Daily average amount of vehicles entering the Seoul metropolitan area highways (N, vehicles) [a)]	1432100.8	1441560.1	1344726.9	1382005.2	1315198.7
Daily average number of citizens who used subways of Seoul Metropolitan area (N, subjects) [b)]	13023.8	13449.0	12424.3	12427.1	9327.4

[a)] Data were retrieved from Korea Transport Database website: https://www.ktdb.go.kr

[b)] Data were retrieved from Seoul open data portal website: http://data.seoul.go.kr

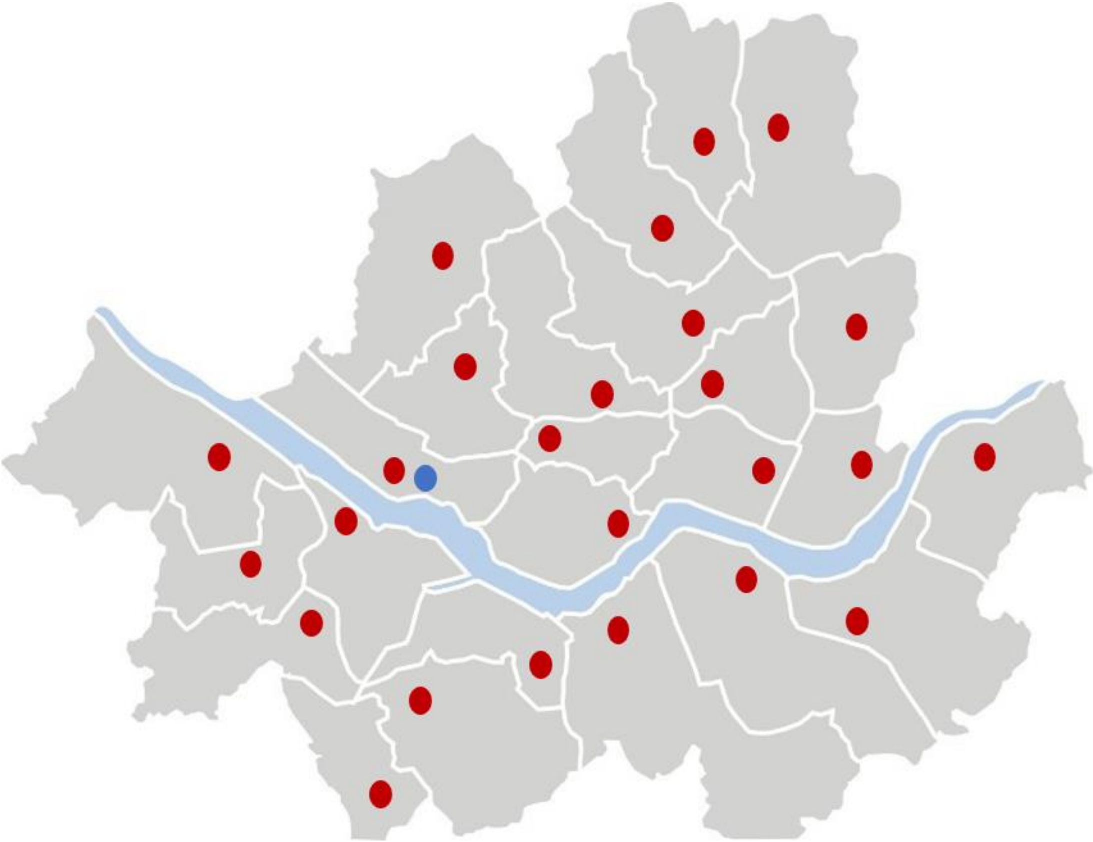

Figure S1. Locations of 25 $PM_{2.5}$ monitoring stations (red circle)

and meteorological station (blue circle) covering Seoul

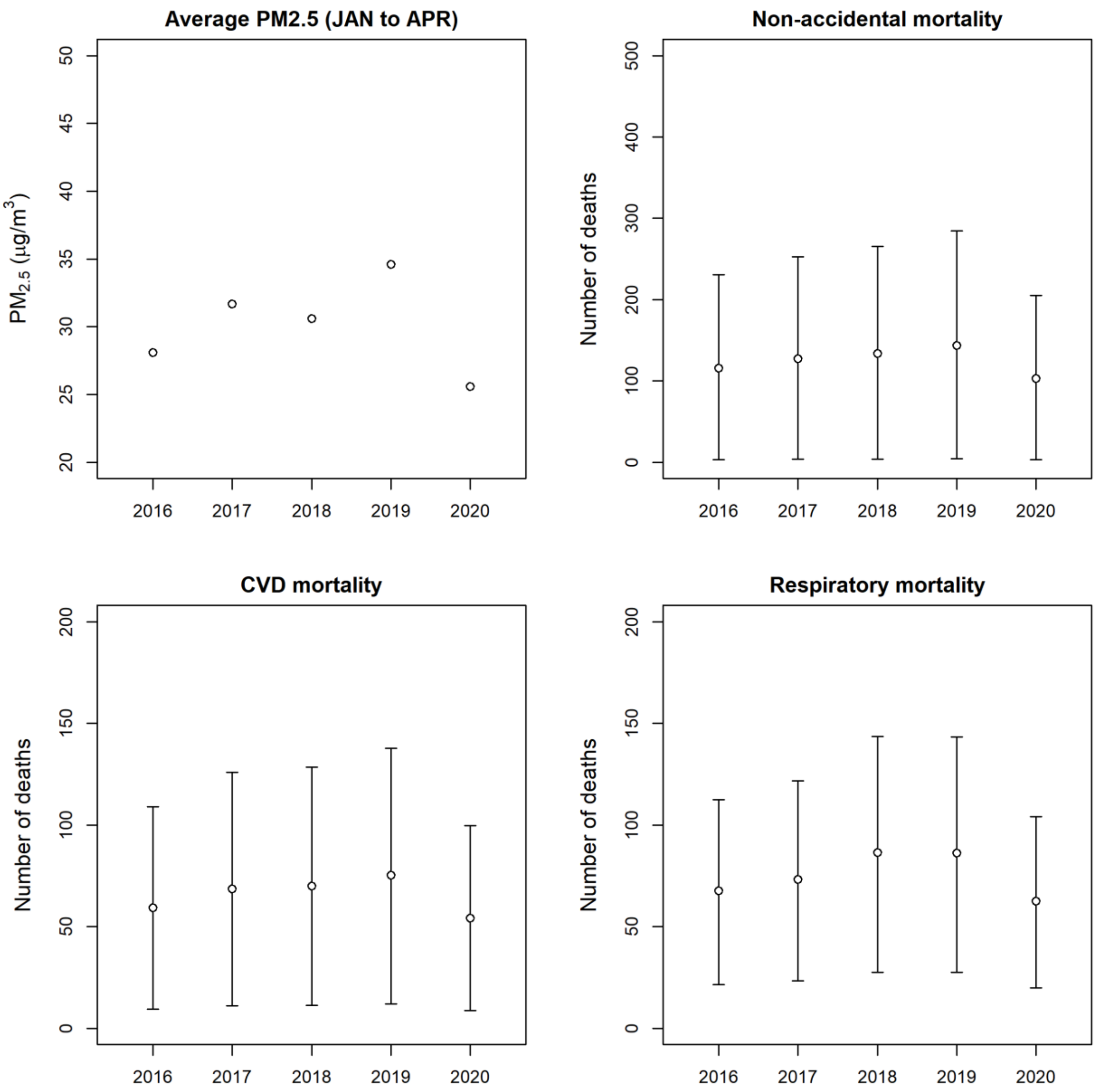

Figure S2. Average $PM_{2.5}$ concentration of Seoul from January to April and estimated number of mortalities attributable to $PM_{2.5}$ exposure (RRs from the Seoul study were used for the estimation)

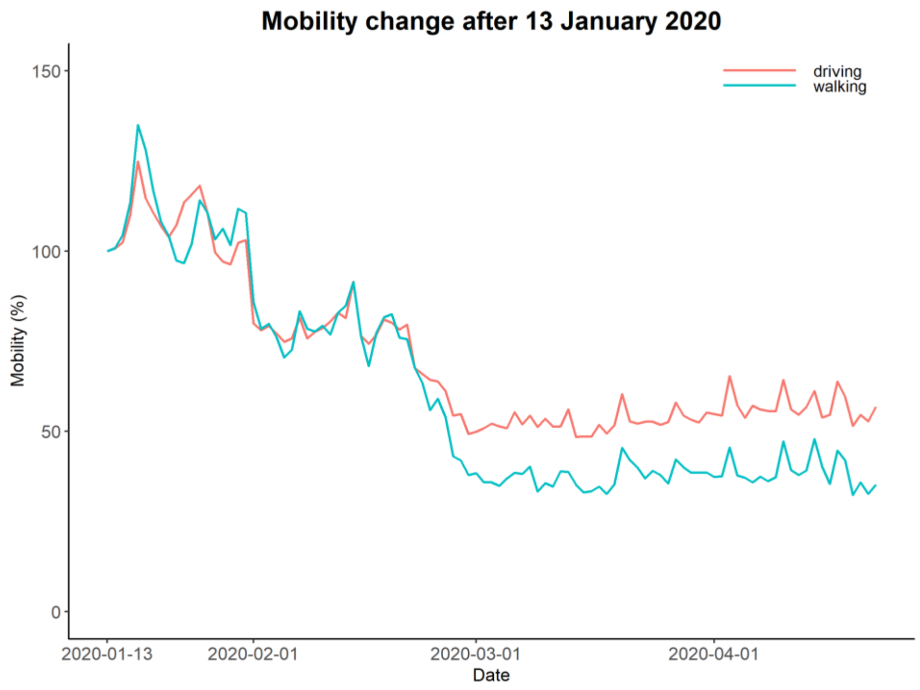

Figure S3. Mobility trends of Seoul City (Mobility on 13 January 2020 was regarded as 100%; Data was gathered from the Apple Maps Mobility Trends Reports: https://www.apple.com/covid19/mobility/)
